# Effects of 1-Deoxynojirimycin Extracts of Mulberry Leaves on Oxidative Stress and the Function of the Intestinal Tract in Broilers Induced by H_2_O_2_

**DOI:** 10.3390/ani14223319

**Published:** 2024-11-18

**Authors:** Chengfeng Zhao, Mingzhu Wang, Tao Li, Dehui Li, Yuan Feng, Yuhua Wang, Liang Qu, Adileidys Ruiz Barcenas, Boris Ramos Serrano, Manman Shen, Weiguo Zhao

**Affiliations:** 1 Jiangsu Key Laboratory of Sericultural Sericulture and Animal Biotechnology, School of Biotechnology, Jiangsu University of Science and Technology, Zhenjiang 212100, China; inmitlessness@163.com (C.Z.); wangmingzhu129@163.com (M.W.); ttaolii@163.com (T.L.); 13603641258@163.com (D.L.); fengyuan6181@126.com (Y.F.); wangyhsri@126.com (Y.W.); 2 Key Laboratory of Silkworm and Mulberry Genetic Improvement, Ministry of Agriculture and Rural Affairs, The Sericultural Research Institute, Chinese Academy of Agricultural Sciences, Zhenjiang 212100, China; 3 Jiangsu Institute of Poultry Science, Chinese Academy of Agricultural Sciences, Yangzhou 225125, China; liangquyz@126.com; 4 Plant Protein and Bionatural Products Research Center, Ministry of Agriculture, Havana 999075, Cuba; adileidys2017@gmail.com (A.R.B.); boris.ramos1972@gmail.com (B.R.S.)

**Keywords:** 1-deoxynojirimycin, intestine, chicken, oxidative stress, intestinal barrier, inflammatory response

## Abstract

The poultry industry struggles with oxidative stress affecting gut health and productivity. This study examined using 1-Deoxynojirimycin (DNJ) extracts from mulberry leaves as an antioxidant in broilers feed to combat this issue. We divided 240 broilers, aged 16 days, into six groups, including a control and groups exposed to oxidative stress through H_2_O_2_ injections, with different supplement levels of DNJ-E (40, 80, 120, and 160 mg/kg of the basal diet) lasting until the broilers reached 42 days old. We evaluated intestinal morphology, ultrastructure, oxidative stress markers, the tight junction, and inflammatory cytokines. Adding 40 mg/kg DNJ-E improved villus height, the villus-to-crypt ratio, and cellular ultrastructure, and increased SOD levels in the jejunum and ileum, as well as CAT levels in the duodenum and jejunum (*p* < 0.05), compared to the H_2_O_2_ group. The addition of DNJ had differential effects on oxidative stress, the intestinal barrier, and immune-related genes. Importantly, the dosages of 40 mg/kg and 80 mg/kg resulted in an upregulation of *MUC2* mRNA expression (*p* < 0.05). These findings suggest that DNJ-E holds potential as a beneficial feed additive for enhancing broiler health, particularly at supplementation levels below 80 mg/kg, as higher concentrations may negatively influence intestinal health. Future investigations should aim to elucidate the underlying mechanisms through which DNJ-E operates within the avian gastrointestinal system.

## Simple Summary

Intestinal health is crucial for safeguarding overall well-being. 1-Deoxynojirimycin (DNJ) from mulberry leaves can reduce oxidative stress and inflammatory responses, ensuring intestinal health. This study evaluated the effects of DNJ extract of mulberry leaves (DNJ-E) as an antioxidant on intestine function in broilers under oxidative stress. The addition of DNJ-E led to improvements in the morphology and ultrastructure of the intestine, as evidenced by increased villus height, an enhanced villus-to-crypt ratio, and strengthened tight junctions. Treatment with 40 mg/kg DNJ-E resulted in elevated levels of SOD and CAT in the jejunum, along with an upregulation of *MUC* mRNA expression. These findings suggest that DNJ-E plays a significant role in enhancing intestinal barrier function by increasing the activity of antioxidant enzymes, thereby contributing to the protection of intestinal health in broilers.

## 1. Introduction

Amidst societal advancement, there is a surging demand for livestock and poultry products, which is driving genetic selection and intensive rearing in the poultry sector. However, this rapid progression has engendered numerous challenges, particularly oxidative stress induced by factors such as temperature, diet, transportation, and the rearing environment [1]. Oxidative stress can diminish immunity, impair performance, and elevate mortality rates in poultry, posing significant threats to the livestock industry [2]. The intestine, a vital organ for nutrient absorption and waste metabolism, plays a crucial role in poultry performance [3]. Yet, it is highly vulnerable to oxidative stress, which can compromise the integrity of the intestinal mucosal barrier and increase its permeability and allow harmful substances to enter the body [4,5]. This susceptibility may result in intestinal inflammation, tissue damage, and impaired repair and regeneration capabilities of intestinal tissues [6]. Oxidative stress can disrupt the balance of beneficial intestinal microbiota, leading to dysbiosis and an elevated risk of intestinal infections and diseases [7]. Therefore, it is essential to investigate the mechanisms underlying intestinal oxidative damage and to enhance research and development efforts focused on potent antioxidant compounds for incorporation into feed formulations.

To cope with the adverse effects of oxidative stress, there has been increasing interest in using natural antioxidants as feed additives to mitigate intestinal damage and promote poultry health [8]. Mulberry leaves, renowned for their rich bioactive compounds, have demonstrated potential in modulating glucose metabolism and exhibiting antioxidant and anti-inflammatory properties [9]. Among these bioactive compounds, 1-Deoxynojirimycin (DNJ), a naturally occurring alkaloid, has been recognized for its ability for glucose-lowering effects [10]. Recent investigations into DNJ have highlighted its notable anti-inflammatory, antiviral, and antitumor properties, as well as its beneficial effects on gastrointestinal health [11,12,13]. Our previous research demonstrated that DNJ enhances antioxidant enzyme activity and mitigates inflammatory responses in the intestinal tract of laying hens [14].Additionally, the antioxidative effects of DNJ have also been observed in the high-glucose-induced oxidative [15] and indomethacin-induced gastric ulcers of mice [16], and in patients suffering from heart disease and blood stasis syndrome [17]. These effects underscore the potential of DNJ in mitigating the intestinal damage induced by oxidative stress and in preserving the overall intestinal health of poultry, which is crucial for the sustainability of the poultry industry. In this study, we utilized H_2_O_2_ to establish an in vivo oxidative stress model in broilers, aiming to investigate the protective effects of DNJ extracted from mulberry leaves (DNJ-E) on intestinal oxidative stress. Our objective was to explore the potential of DNJ-E as an effective dietary antioxidant in feed supplementation, thereby enhancing the poultry industry’s approach for mitigating the detrimental effects of oxidative stress on intestine health.

## 2. Materials and Methods

### 2.1. Experimental Design, Animals, and Sampling

#### 2.1.1. Experimental Design and Animals

**Figure 1:**
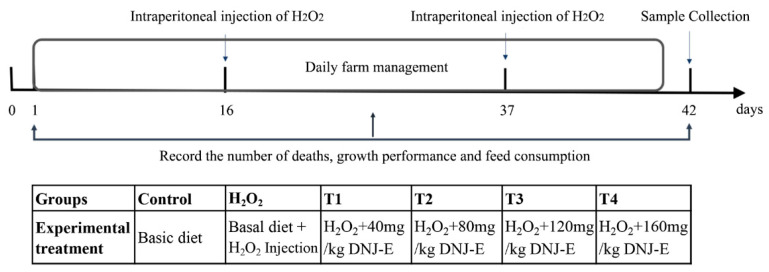
Oxidative stress model flow chart and experimental groups of broilers.

The animal experiments were approved by the Institutional Animal Care and Use Committee of Jiangsu University of Science and Technology (Zhenjiang, China, approval number GSB202131002). A total of 240 one-day-old healthy AA broilers were obtained from Jiangsu Jianghai Poultry Co., Ltd. (Nantong, Jiangsu, China). The basal diet formulations and nutrient levels were designed according to the guidelines of the NRC (1994) [18] and are detailed in Table 1. At the age of 16 days old, the broilers were randomly assigned to six groups, each comprising eight replicates with five birds per replicate. The groups were as follows: the control group, which received a basal diet and intraperitoneal injections of 0.75% saline (Kelun, Chengdu, China); the H_2_O_2_ group, which was fed a basal diet and received intraperitoneal injections of a 2.96 mM/kg BW H_2_O_2_ solution (Sinopharm, Beijing, China); and the groups supplemented with DNJ extracts from mulberry leaves (DNJ-E). These groups also received intraperitoneal injections of H_2_O_2_ as in the H_2_O_2_ group but were fed a basal diet supplemented with DNJ-E at varying levels of 40 (T1), 80 (T2), 120 (T3), and 160 (T4) mg/kg DNJ-E (50% purity; Shengqing, Xi’an, China). The experimental duration was 42 days, during which intraperitoneal injections of H_2_O_2_ were administered on days 16 and 37 following the protocol of a previous study [19]. The design of the trial and the workflow are shown in Figure 1. Broilers were reared according to the feeding standards of China for broilers (NY/T33-2004) [20], ensuring uniform lighting and vaccination procedures. The broilers were housed in individual cages for each replicate from 1 to 14 days of age and were subsequently transferred to individual cages, with one bird per cage, after day 14. All birds had ad libitum access to nipple-type drinkers for feed and water. The environmental temperature was maintained at 32 °C for the initial three days and was gradually reduced by 2–3 °C each week, reaching 20 °C until the end of the trial.

#### 2.1.2. Sample Collection

At the end of the 42-day feeding trial, one chicken from each replicate was selected close to the average body weight assessment, resulting in a total of eight chickens per group being euthanized for analysis. After a 12 h fasting period, the chickens were euthanized by cervical dislocation. The abdominal cavity was opened to collect samples of duodenum, jejunum, and ileum. Portions of these tissues were fixed in 4% paraformaldehyde (Sangon Biotech, Shanghai, China) for subsequent histological examination, while other portions were placed in cryogenic vials, snap-frozen in liquid nitrogen, and stored at −80 °C for future gene expression analysis. For the jejunum, samples were rapidly harvested and fixed in glutaraldehyde to prevent mechanical damage prior to transmission electron microscopy analysis.

### 2.2. Observation of Intestinal Morphology

#### 2.2.1. Hematoxylin–Eosin Stain

Duodenal, jejunal, and ileal tissues were fixed in 4% paraformaldehyde, dehydrated through a graded series of ethanol, embedded in paraffin, sectioned, and subsequently deparaffinized using xylene and an ethanol gradient. The sections were stained with hematoxylin–eosin (HE) and mounted for analysis. Microscopic imaging was conducted using a microscope (IX73, Olympus, Tokyo, Japan) to measure the villus height and crypt depth in each sample using a computer-assisted morphometric system. Villus height was defined as the distance from the tip to the base of the villi, while crypt depth was measured from the base of the villi to the base of the crypts. The villus-to-crypt ratio was calculated as the ratio of villus height to crypt depth.

#### 2.2.2. Transmission Electron Microscopy

To observe ultrastructure morphology, transmission electron microscopy (TEM; FEI Tecnai F20, Thermo, Hillsboro, OR, USA) was performed following a meticulous protocol. Post-fixation was carried out using osmium tetroxide (OsO4) in a dark chamber, followed by thorough rinsing with phosphate buffer (PBS). Dehydration was conducted through using a graded series of ethanol, followed by two changes of acetone, each lasting 15 min. Infiltration and embedding were performed with a mixture of acetone and epoxy resin (812 embedding agent), followed by pure resin infiltration and overnight incubation in an embedding mold at 37 °C. The embedded samples were then polymerized at 65 °C for over 48 h. Ultrathin sections, measuring 60–80 nm, were prepared and collected on copper grids with uranyl acetate and lead citrate. After staining, the grids were washed, dried, and then observed under a TEM for image acquisition and analysis.

### 2.3. Antioxidative Enzymes and Inflammatory Cytokines Assays

An enzyme-linked immunosorbent assay (ELISA) was employed to measure the oxidative stress markers of total superoxide dismutase (SOD) and catalase (CAT) in duodenal, jejunal, and ileal tissues. Tissue samples were homogenized in ice-cold saline using a glass homogenizer at a ratio of 1:10 (*w*/*v*). The homogenates were subsequently analyzed spectrophotometrically according to the protocols provided with the ELISA kits obtained from Nanjing Jiancheng Bioengineering Institute (Nanjing, China). Briefly, to 1 g of intestinal tissues 9 mL of physiological saline was added to shear the tissue in an ice-water bath. The resulting 10% homogenate was centrifuged, and the supernatant was mixed with the working solution according to the manufacturer’s instructions. Absorbance readings were taken using a microplate reader (BioTek Epoch 2, Agilent, San Jose, CA, USA) at wavelengths of 450 and 405 nm. All analyses were conducted in triplicate.

### 2.4. Quantitative Reverse Transcription of PCR (qRT-PCR)

Total RNA was isolated from tissue samples using Trizol reagent from Vazyme Co., Ltd. (Nanjing, China) in accordance with the manufacturer’s instructions. The integrity and concentration of RNA were evaluated using a NanoDrop spectrophotometer (Implen, München, Germany), with samples exhibiting a 260/280 nm ratio within the range of 1.8 to 2.0 before proceeding to subsequent analyses. The synthesis of cDNA was conducted by HiScript II Q Select RT SuperMix (Vazyme, Nanjing, China), strictly following the manufacturer’s recommendations. The qRT-PCR analysis was performed utilizing the ChamQ SYBR Color qPCR Master Mix Kit (Vazyme, Nanjing, China) on a CFX96 Real-Time PCR detection system (Bio-Rad, Hercules, CA, USA). The reaction system comprised 10 µL of 2× ChamQ SYBR Color qPCR Master Mix, 0.4 µL each of the forward and reverse primers at a concentration of 10 µM, 1.0 µL of cDNA, and nuclease-free water to a final volume of 20 µL. The PCR protocol included an initial denaturation step at 95 °C for 30 s, followed by 40 cycles of denaturation at 95 °C for 10 s, and annealing/extension at 60 °C for 30 s, concluding with a melt curve analysis ranging from 60 to 95 °C. The expression levels of genes associated with oxidative stress and inflammatory responses were normalized against β-actin (*ACTB*) as an endogenous control. Primer selection was based on high amplification efficiency, with only those exhibiting greater than 90% efficiency being included in this study. A list of all primer sequences is provided in Table 2. The 2^−ΔΔCT^ method was employed to calculate the relative quantification of mRNA expression.

### 2.5. Statistical Analyses

Data distribution was assessed using the Kruskal–Wallis test to evaluate normality. Data that conformed to a normal distribution were analyzed using one-way ANOVA. Results are expressed as the mean ± standard error of the mean (SEM). Statistical analyses were conducted using SPSS version 20.0 (IBM SPSS, Armonk, NY, USA). Post hoc mean comparisons were performed using the least significant difference (LSD) test, with a significance threshold set at *p* < 0.05. Data visualization was generated using GraphPad Prism version 8.0.1 (San Diego, CA, USA).

## 3. Results

### 3.1. Effects of DNJ-E on Intestinal Morphology of Broilers Under Oxidative Stress Conditions

**Figure 2:**
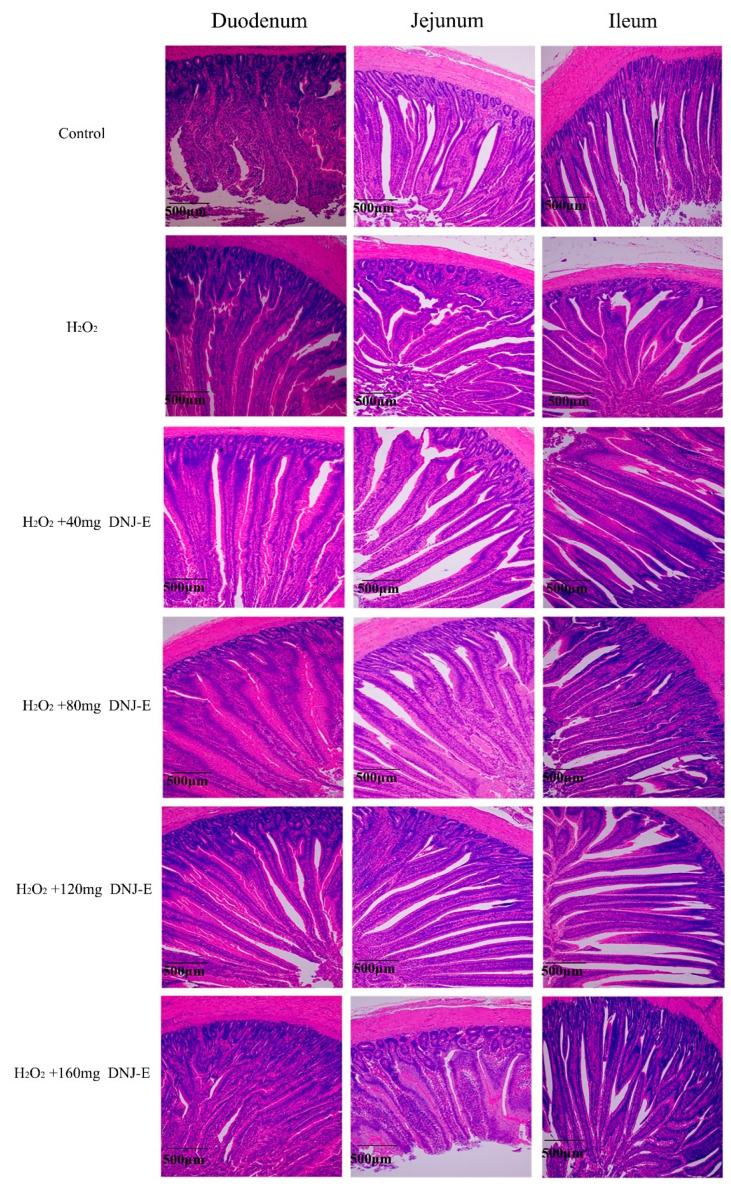
Effects of dietary supplementation of DNJ-E on intestinal morphological changes in broilers under oxidative stress model.

As illustrated in Figure 2 and detailed in Table 3, treatment with H_2_O_2_ caused a decrease in villus height in the duodenum and jejunum and an increase in crypt depth in the duodenum (*p* < 0.05), compared to the control group. Conversely, the supplementation of DNJ-E at various concentrations in the H_2_O_2_-treated groups mitigated these effects, leading to restored villus length, decreased crypt depth, and an improved villus-to-crypt ratio. Notably, a dosage of 40 mg/kg DNJ-E proved particularly effective in the duodenum and ileum (*p* < 0.05), while 120 mg/kg DNJ-E exhibited the highest efficacy in the jejunum (*p* < 0.05).

**Figure 3:**
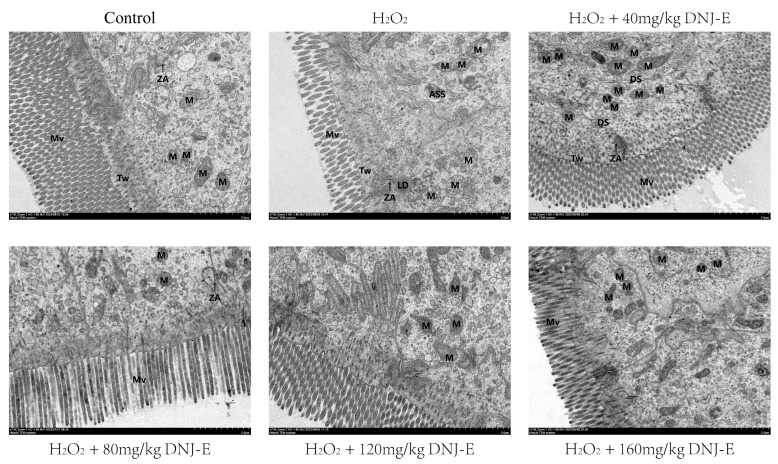
The intestinal ultrastructure of broilers under oxidative stress models by adding DNJ-E to the diet. Note: Mv: microvillus; ZA: Zonula adherents; Tw: Terminal web; M: mitochondria; ASS: Autolysosome; LDs: Lipid droplets DS: Desmosome. Scale: 2.0 μm.

Figure 3 illustrates the effects of DNJ-E on the intestinal ultrastructure of broilers. The microvilli in the H_2_O_2_ group appear shorter compared to those in the control group. Following H_2_O_2_ treatment, ultrastructure alterations include less distinct tight junctions between epithelial cells, which exhibit widened intermediate junction gaps and vague bridging granules, indicating damage to the intestinal barrier and a slight increase in cellular gaps. Mitochondrial changes are characterized by a disrupted matrix, sparse distribution, and alterations to the cristae, which are either fragmented, shortened, or reduced. Additionally, the rough endoplasmic reticulum exhibits dilation and vesiculation, along with considerable degranulation of ribosomes. In contrast, the intestinal ultrastructure in the DNJ-E group shows a restoration of the microvillar architecture, characterized by tightly arranged and elongated microvilli. The tight junctions are more pronounced, and the intercellular gaps are narrower. The mitochondrial matrix and membrane structures remain intact. Notably, the group receiving 40 mg/kg DNJ-E demonstrated the most significant improvements, suggesting that this concentration was the most effective in mitigating the adverse effects induced by H_2_O_2_ treatment.

### 3.2. Effects of DNJ-E on Intestinal Oxidative Indices of Broilers Under Oxidative Stress State

**Figure 4:**
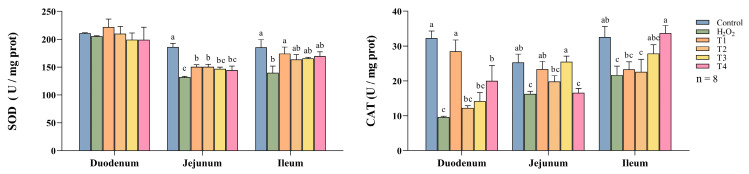
Effects of dietary supplementation of DNJ-E on intestinal oxidation indexes in broilers under oxidative stress model. Note: ^a–c^: different characters on the column represent differences (*p* < 0.05). Control: basic diet group; H_2_O_2_: H_2_O_2_ group (intraperitoneal injection of 2.96 mmol/kg BW H_2_O_2_ solution.); T1: H_2_O_2_ + 40 mg/kg DNJ-E group; T2: H_2_O_2_ + 80 mg/kg DNJ-E group; T3: H_2_O_2_ + 120 mg/kg DNJ-E group; T4: H_2_O_2_ + 160 mg/kg DNJ-E group. The same as below.

Figure 4 delineates the impact of DNJ-E on the activity of antioxidant enzymes in the intestinal tract of H_2_O_2_-treated broilers. The administration of H_2_O_2_ led to a significant reduction in SOD levels within the jejunum and ileum, and in CAT levels across all intestinal segments (*p* < 0.05). The inclusion of 40 mg/kg DNJ-E in the diet significantly elevated the levels of SOD and CAT in the jejunum, as well as SOD in the ileum and CAT in the duodenum, beyond those in the H_2_O_2_-treated group (*p* < 0.05). Furthermore, compared to the H_2_O_2_-treated group, 80 mg/kg DNJ-E increased SOD activity in the jejunum (*p* < 0.05), 160 mg/kg DNJ-E increased CAT levels in the duodenum and ileum (*p* < 0.05), and 40 mg/kg DNJ-E increased CAT levels in the duodenum. These findings highlight DNJ-E’s strong enhancement of antioxidant defenses, with the 40 mg/kg dose proving the most effective.

### 3.3. Effects of DNJ-E on the Expression of Intestinal Tight Junction-Related Genes in Broilers Under Oxidative Stress State

**Figure 5:**
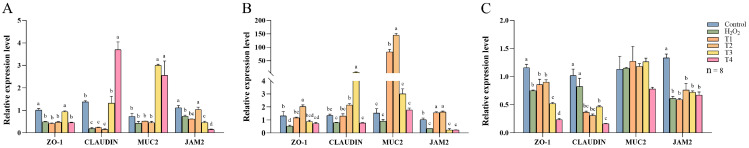
Effects of dietary supplementation of DNJ-E on the expression levels of intestinal tight junction-related genes in broilers under oxidative stress model. Note: ^a–d^: different characters on the column represent differences (*p* < 0.05). (**A**) Duodenum; (**B**) jejunum; (**C**) ileum.

Figure 5 illustrates the effects of H_2_O_2_ treatment and DNJ-E supplementation on the expression of genes essential for intestinal barrier function. In the duodenum, H_2_O_2_ treatment resulted in a significant reduction in *ZO-1*, *CLAUDIN*, and *JAM2* mRNA levels (*p* < 0.05). However, the addition of 120 mg/kg DNJ-E to the diet reversed the effects on *ZO-1* and *CLAUDIN* (*p* < 0.05), while treatment with 160 mg/kg DNJ-E improved *CLAUDIN* and MUC2 mRNA levels (*p* < 0.05). In the jejunum, supplementation with 40 and 80 mg/kg DNJ-E mitigated the H_2_O_2_-induced reductions in *ZO-1*, *CLAUDIN*, *JAM2*, and *MUC2* mRNA levels. Notably, *MUC2* mRNA levels increased dramatically—by several orders of magnitude (*p* < 0.05, Figure 5C). In the ileum, while H_2_O_2_ decreased *ZO-1* and *JAM2* mRNA, supplementation with 40 mg/kg and 80 mg/kg DNJ-E increased *ZO-1* mRNA levels, although this increase did not achieve statistical significance (*p* > 0.05, Figure 5C).

### 3.4. Effects of DNJ-E on the Expression of Genes Related to Oxidation and Inflammation in the Intestinal Tract of Broilers Under Oxidative Stress State

**Figure 6:**
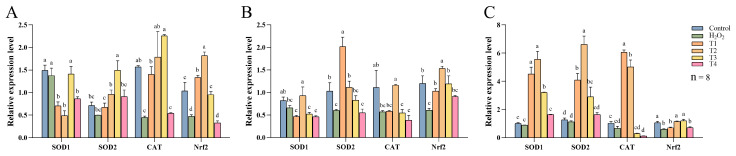
Effects of dietary supplementation of DNJ-E on the expression levels of intestinal oxidation-related genes in broilers under oxidative stress model. Note: ^a–d^: different characters on the column represent significant differences (*p* < 0.05). (**A**) Duodenum; (**B**) jejunum; (**C**) ileum.

Figure 6 illustrates the effects of DNJ-E on the mRNA expression of antioxidant genes across different segments of the broiler intestine following H_2_O_2_ treatment. In the duodenum, the supplementation of 120 mg/kg DNJ-E significantly upregulated *SOD2* mRNA expression levels, while *CAT* and *Nrf2* mRNA levels were elevated in the 40, 80, and 120 mg/kg DNJ-E concentrations compared to the H_2_O_2_-treated group (Figure 6A, *p* < 0.05). In the jejunum, an 80 mg/kg dose of DNJ-E significantly increased mRNA levels of *SOD1*, *CAT*, and *Nrf2* compared to the H_2_O_2_-treated group (*p* < 0.05, compared to the H_2_O_2_-treated group). Additionally, *SOD2* mRNA expression was also elevated with 40 and 80 mg/kg DNJ-E supplementation relative to the H_2_O_2_-treated group (Figure 6B, *p* < 0.05). In the ileum, *SOD1* and *SOD2* mRNA expression levels showed significant increase at 40, 80, and 120 mg/kg DNJ-E concentrations when compared with the H_2_O_2_-treated group (*p* < 0.05), while *CAT* mRNA expression was elevated at 40 and 80 mg/kg DNJ-E supplementation (*p* < 0.05). Notably, *SOD1*, *SOD2*, and *CAT* mRNA levels peaked at the 40 mg/kg and 80 mg/kg DNJ-E doses. Furthermore, *Nrf2* mRNA levels were significantly reduced in all the intestinal segments following H_2_O_2_ treatment; however, Nrf2 mRNA levels in the duodenum increased in the groups treated with 40, 80, and 120 mg/kg DNJ-E, and ileal Nrf2 levels were elevated at 80 and 120 mg/kg DNJ-E supplementation compared to the H_2_O_2_-treated group (*p* < 0.05).

**Figure 7:**
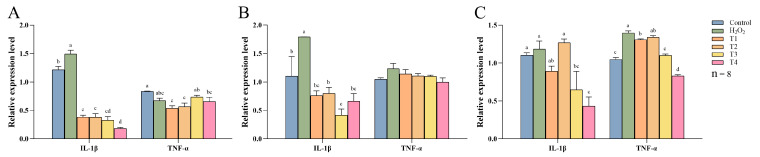
Effects of dietary supplementation of DNJ-E on the expression levels of intestinal inflammation-related genes in broilers under oxidative stress model. Note: ^a–d^: different characters on the column represent significant differences (*p* < 0.05). (**A**) Duodenum; (**B**) jejunum; (**C**) ileum.

Figure 7 illustrates the effects of DNJ-E on the mRNA expression of inflammatory cytokines *IL-1β* and *TNF-α* in the intestine following H_2_O_2_ treatment. H_2_O_2_ significantly upregulated *IL-1β* mRNA levels in both the duodenum and jejunum compared to the control group (*p* < 0.05). In contrast, supplementation with various concentrations of DNJ-E resulted in a significant reduction in *IL-1β* mRNA expression both in the duodenum and jejunum compared to the H_2_O_2_-treated group (*p* < 0.05). Regarding the *TNF-α* mRNA levels in the duodenum, a significant decrease was observed in the 40 and 80 mg/kg DNJ-E groups compared to the control group (*p* < 0.05). However, no significant difference was observed when compared to the H_2_O_2_-treated group. In the jejunum, *TNF-α* mRNA expression levels did not exhibit significant changes following either H_2_O_2_ or DNJ-E treatment compared to the control group (*p* > 0.05). In the ileum, the addition of 120 and 160 mg/kg DNJ-E led to a significant decrease in both *IL-1β* and *TNF-α* mRNA expression levels compared to the H_2_O_2_-treated group (*p* < 0.05).

## 4. Discussion

Oxidative stress significantly impacts poultry health, particularly affecting intestinal functionality and nutrient absorption [21,22]. Preserving intestinal integrity is crucial for poultry well-being. Recent research indicates that plant-derived additives [23,24,25,26] like DNJ, a potent α-glucosidase inhibitor found in mulberry leaves, can enhance antioxidant defenses and immune responses, thereby mitigating oxidative stress and promoting overall health [11,14,16]. As common indicators for estimating intestinal integrity, the villus height, crypt depth, and the villus/crypt ratio can reveal some information on gut health in poultry. The crypts, situated between the intestinal villi, serve as the generative sites for enterochromaffin cells within the mucosal layer. A shallower crypt depth facilitates villus regeneration and supports optimal growth conditions, while an intact crypt structure is essential for maintaining mucosal stability and enhancing the integrity of the mucosal barrier [27]. Our findings demonstrated that dietary inclusion of DNJ-E at doses below 80 mg/kg effectively mitigates the intestine injury induced by H_2_O_2,_ as evidenced by improvements in the villus height and villus/crypt ratio indicators. Previous research has indicated that DNJ can modulate the intestinal microbiota [28,29], stimulate autophagy for cellular repair and regeneration [30], and enhance the absorption and tissue distribution of nitrogenous sugars in the gut, thereby contributing to its bioavailability and bioactivity [31,32].

Intestinal barrier function is maintained by a complex system on the intestinal mucosal surface, comprising the mucus layer, epithelial cells, tight junctions, and the bridge particles between intestinal epithelial cells. This synergistic array of structures and functions protects the intestinal tract from harmful substances and ensures nutrient absorption [33]. Our ultrastructural appearance analysis revealed that DNJ-E treatment improved both mitochondrial and tight junction integrity in the intestines. Mitochondria, as the primary sites of energy production within cells, play a crucial role in cellular respiration by efficiently neutralizing ROS generated during metabolic processes. By regulating redox balance, mitochondria prevent the excessive generation and accumulation of oxygen free radicals, thereby protecting cells from oxidative stress [34]. Furthermore, previous study has confirmed that DNJ influences cardiac function through binding to the OPA, a protein necessary for mitochondrial fusion [35]. Zheng et al. [29] discovered that DNJ could mitigate high-fat diet-induced nonalcoholic steatohepatitis by balancing intestinal microbial composition. However, additional research is needed to explore whether DNJ influences mitochondrial function and subsequently affects intestinal barrier integrity in poultry by interacting with OPA1.

*ZO-1*, *CLAUDIN*, *MUC2*, and *JAM2* are essential genes that are vital for maintaining intestinal health, as they play significant roles in preserving the integrity of the intestinal barrier and preventing the onset of disease [36]. Among them, MUC2 is a mucin secreted by cup cells. They form a mucus barrier that protects the gut from microorganisms, pathogens, and toxins and maintains cell polarity, and thus play a key role in regulating paracellular permeability and intercellular adhesion [37,38]. Our findings indicate that supplementing the diet with DNJ-E at specific concentrations in the duodenum, jejunum, and ileum effectively upregulated the mRNA expression of these key genes, where the inclusion of 40 and 80 mg/kg DNJ-E in the diet greatly increased MUC2 mRNA expression in the jejunum. It is hypothesized that DNJ-E may enhance MUC2 expression by modulating signaling pathways within goblet cells, such as the NF-κB or MAPK pathways [39,40]; however, the protective role of DNJ in the intestinal barrier warrants further exploration into its precise mechanisms.

Antioxidant enzymes, including SOD and CAT, are pivotal in cellular defense mechanisms against oxidative stress. These enzymes protect cellular integrity by neutralizing free radicals and other reactive oxygen species through catalytic reduction reactions, thereby diminishing their concentrations and mitigating oxidative damage [41]. Our findings suggest that dietary supplementation with DNJ-E at optimal levels can effectively combat oxidative stress in the intestines. Specifically, DNJ-E restored CAT levels in the duodenum and increased both SOD and CAT in the jejunum at a 40 mg/kg concentration. The ileum exhibited an elevation in SOD levels at the 40 mg/kg dose and an enhancement in CAT levels at a higher dose of 160 mg/kg. The unchanged SOD levels in the duodenum may reflect minimal variations among groups. Furthermore, our research demonstrates that the antioxidant effectiveness of DNJ is influenced by the specific intestinal site, likely owing to the distinct structural and functional characteristics of the duodenum, jejunum, and ileum [42,43]. Differences in pH levels, microbial composition, and permeability across these segments may impact the responsiveness epithelial cells to DNJ, thereby affecting its stability and absorption within the gastrointestinal tract [42]. Oxidative stress indices further confirmed the antioxidant effects of DNJ, with varying efficacies observed across different intestinal sites. The qRT-PCR analysis revealed that varying dietary supplementation with DNJ-E increases the different expression levels of genes related to oxidative stress including *SOD1*, *SOD2*, *CAT*, and *Nrf2* in broilers’ intestines. *Nrf2*, as a key regulator of the *Keap1/Nrf2* signaling pathway, is crucial for maintaining cellular homeostasis and defending against oxidative stress. It achieves this by binding to the antioxidant response element (ARE) and modulating the expression of various antioxidant enzymes, such as SOD and CAT, and anti-inflammatory cytokines [44,45,46]. Our previous study confirmed that DNJ elevates the *Nrf2* mRNA levels in intestinal epithelial cells cultured in vitro [14]. The present result further supports the hypothesis that DNJ activates the Keap1/Nrf2 signaling pathway, thereby regulating oxidative stress in the broiler gastrointestinal tract.

*IL-1β* and *TNF-α*, key pro-inflammatory cytokines, are instrumental in modulating immune reactions and promoting cell demise by facilitating the recruitment, activation, and migration of inflammatory cells and stimulating the production of additional cytokines, which amplify the inflammatory response [47,48]. The reduction in *IL-1β* in the duodenum and jejunum upon DNJ-E treatment in all concentrations illustrates the better action of DNJ in anti-inflammatory effects in the intestine.

Our study reveals that DNJ exhibits variable effects on the expression of genes related to the intestinal barrier, oxidative stress, and immunity, depending on its concentration. This nonlinear response may arise from DNJ’s differential modulation of signaling pathways at different concentration levels. Comparable findings have been reported in previous studies conducted in different cellular or animal models. For instance, DNJ derivatives at concentrations of 5 μM and 10 μM have been shown to induce DNA damage, mitochondrial dysfunction, and oxidative stress in HCT-16 cells [49]. Oral administration of 100 mg/kg/day DNJ significantly mitigated oxidative stress-related injury in septic cardiomyopathy in mice, an effect mediated through the JAK2/STAT6 signaling pathway [11]. Treatment of HUNEC cells with 5 μM DNJ reduced high-glucose-induced oxidative stress through the NRF2/OGG1 signaling pathway [15]. In our previous study involving laying hens, supplementation with DNJ below 100 mg/kg DNJ to the basal diet enhanced the activity of antioxidant enzymes [14]. These findings suggest that DNJ, at low or high concentrations, may activate or inhibit distinct signaling pathways, leading to nonlinear changes in the gene expression. This concentration-dependent effect is an important consideration in poultry nutrition research, as it may impact intestinal health, antioxidant capacity, and immune response. However, for the intestine structure, concentrations of DNJ-E below 80 mg/kg exhibit a better anti-oxidative stress effect.

## 5. Conclusions

The findings of this study indicate that DNJ-E holds potential as a valuable antioxidant feed additive. The inclusion of various concentrations, especially less than 80 mg/kg of DNJ-E, in the diet significantly enhanced the recovery of intestinal villus height, crypt depth, and the villus-to-crypt ratio in broilers experiencing oxidative stress, thereby enhancing intestinal barrier function. Notably, supplementing the basal diet with 40 mg/kg of DNJ-E mitigated oxidative stress-induced intestinal damage by elevating the activities of the antioxidant enzymes SOD and CAT, while upregulating the expression levels of MUC mRNA and decreasing the expression levels of pro-inflammatory cytokines *IL-1β* and *TNF-α*. This multifaceted action contributes to the preservation of intestinal health in broilers. Although these findings are promising, future research should investigate the mechanisms by which low doses of DNJ influence antioxidant pathways and signaling cascades. Understanding these actions could inform the application of other plant-derived bioactive compounds in poultry nutrition, enhancing gut health and overall performance in various production systems.

## Figures and Tables

**Table 1:** Ingredient composition and nutrient levels of experimental diets (% Dry matter).

Items	1–21 Days	22–24 Days
**Ingredients (%)**		
Maize	59.00	59.70
Soybean Meal ^2^	35.10	33.01
Soybean Oil	2.00	4.00
Limestone	1.48	1.59
Dicalcium Phosphate	1.66	1.27
Methionine	0.20	0.10
Lysine	0.13	0.03
Cysteine	0.30	0.33
Salt	0.30	0.30
Premix ^3^	0.60	0.60
**Total**	100.00	100.00
**Nutrient Levels (%) ^1^**		
Metabolisable Energy (MJ/kg)	12.56	12.52
Crude Protein	21.10	19.60
Cysteine	0.85	0.76
Methionine	0.50	0.42
Lysine	1.20	1.05
Available Phosphorus	0.46	0.39
Total Calcium	1.00	0.95

Notes: Values are expressed on an air-dried basis. ^1^: Calculated values. ^2^: Soybean meal contains 44% crude protein. ^3^: Per kg of premix provides Vitamin A, 54,000,000 IU; Vitamin D3, 10,800,000 IU; Vitamin E, 15,000 mg; Vitamin K3, 5000 mg; Vitamin B1, 2000 mg; Vitamin B2, 15,000 mg; Vitamin B12, 65 mg; Pantothenic Acid, 25,000 mg; Biotin, 500 mg; Vitamin C, 50,000 mg; Folic Acid, 1000 mg; Niacin, 15,000 mg; Body Condition Enhancer, 15,000 mg; Methionine Hydroxy Analog, 15,000 mg; Lysine, 1000 mg; Organic Manganese/Zinc/Copper/Iron, etc., 200 mg.

**Table 2:** Primer sequences used in this study.

Gene Name	Primer Sequence (5′-3′)	Product Length (bp)	Accession No.
*ACTB*	F: CAGCCATCTTTCTTGGGTAT R: CTGTGATCTCCTTCTGCATCC	167	NM_205518.1
*ZO-1*	F: TGACTCTTCACAGGGCTCCT R: GGCCTCCTTTCAGCACATCA	120	XM_046925214.1
*CLDN1*	F: CTGGGTCTGGTTGGTGTGTT R: GGTGTTAACAGGTGTGAAAGGG	204	NM_001013611.2
*MUC2*	F: GACATGTGGTCTCTGTGGGG R: GCAGAGCCCGAGTTTCATCA	155	XM_040673077.2
*JAM2*	F: GGCTATTCTTAGTTGCAAGCACA R: CTCTTCGCTCTTCGCACTGA	223	XM_046907882.1
*SOD1*	F: TTGTCTGATGGAGATCATGGCTTC R: TGCTTGCCTTCAGGATTAAAGTGAG	98	NM_205064
*SOD2*	F: AGAGGAGAAATACAAAGAGGCG R: AGCCTGATCCTTGAACACCA	245	NM_204211.2
*CAT*	F: TGCAAGGCGAAAGTGTTTGA R: CCCACAAGATCCCAGTTACCT	158	NM_001031215.2
*Nrf2*	F: TGACCCAGTCTTCATTTCTGC R: GGGCTCGTGATTGTGCTTAC	186	XM_046921130.1
*IL-1β*	F: CCTCCAGCCAGAAAGTGAGG R: TTGTAGCCCTTGATGCCCAG	109	NM_204524.2
*TNF-α*	F: ATCCTCACCCCTACCCTGTC R: TGTTGGCATAGGCTGTCCTG	92	XM_046927265.1

Note: *ACTB*, Actin Beta; *ZO-1*, Zona Occludens 1; *CLDN1*, Claudin-1; *MUC2*, Mucin 2; *JAM2*, Junctional adhesion molecule 2; *SOD1*, superoxide dismutase 1, soluble; *SOD2*, superoxide dismutase 2, soluble; *CAT*, catalase; *Nrf2*, Nuclear Factor (Erythroid-Derived 2)-Like; *IL-1β*, interleukin 1, beta; *TNF-α*, tumor necrosis factor-alpha.

**Table 3:** Quantitative results of HE staining of intestinal tissue morphology.

Organs	Items (μm)	Control	H_2_O_2_	DNJ-E Addition Level (mg/kg)				Standard Error	*p*-Value
				40	80	120	160		
Duodenum	Villus Height	1450.3 ^b^	1291.5 ^c^	1780.6 ^a^	1425.4 ^bc^	1446.4 ^b^	1418.9 ^bc^	25.123	<0.001
	Crypt Depth	109.2 ^b^	133.0 ^a^	86.50 ^c^	80.20 ^c^	81.00 ^c^	84.2 ^c^	3.510	<0.001
	Villus-to-Crypt Ratio	14.9 ^b^	9.9 ^c^	21.0 ^a^	16.6 ^b^	17.8 ^b^	17.5 ^b^	0.590	<0.001
Jejunum	Villus Height	1250.3 ^cd^	1093.0 ^e^	1403.0 ^b^	1366.2 ^bc^	1540.1 ^a^	1195.1 ^de^	23.620	<0.001
	Crypt Depth	82.4 ^ab^	93.8 ^a^	80.9 ^b^	85.4 ^ab^	87.0 ^ab^	80.7 ^b^	1.457	0.084
	Villus-to-Crypt Ratio	15.61 ^ab^	11.8 ^c^	16.8 ^ab^	16.2 ^ab^	18.0 ^a^	15.0 ^b^	0.401	<0.001
Ileum	Villus Height	879.7 ^c^	705.8 ^c^	1455.9 ^a^	1060.7 ^b^	1112.8 ^b^	1081.4 ^b^	33.212	<0.001
	Crypt Depth	78.7 ^ab^	79.2 ^ab^	72.8 ^bc^	78.1 ^ab^	66.3 ^c^	82.3 ^a^	1.285	0.001
	Villus-to-Crypt Ratio	11.3 ^de^	9.3 ^e^	20.3 ^a^	13.6 ^cd^	16.7 ^b^	14.1 ^c^	0.590	<0.001

Notes: ^a–e^ means significant difference between groups (*p* < 0.05).

## Data Availability

Data are contained within this article.

## References

[1] Kpomasse C.C., Oke O.E., Houndonougbo F.M., Tona K. (2021). Broilers production challenges in the tropics: A review. Vet. Med. Sci..

[2] Oke O.E., Akosile O.A., Oni A.I., Opowoye I.O., Ishola C.A., Adebiyi J.O., Odeyemi A.J., Adjei-Mensah B., Uyanga V.A., Abioja M.O. (2024). Oxidative stress in poultry production. Poult. Sci..

[3] Aruwa C.E., Pillay C., Nyaga M.M., Sabiu S. (2021). Poultry gut health–microbiome functions, environmental impacts, microbiome engineering and advancements in characterization technologies. J. Anim. Sci. Biotechnol..

[4] Liu P.Y., Wang Y.B., Yang G., Zhang Q.H., Meng L.B., Xin Y., Jiang X. (2021). The role of short-chain fatty acids in intestinal barrier function, inflammation, oxidative stress, and colonic carcinogenesis. Pharmacol. Res..

[5] Wen X., Tang L., Zhong R., Liu L., Chen L., Zhang H. (2023). Role of Mitophagy in Regulating Intestinal Oxidative Damage. Antioxidants.

[6] Akbar A., Walters J.R.F., Ghosh S. (2009). Review article: Visceral hypersensitivity in irritable bowel syndrome: Molecular mechanisms and therapeutic agents. Aliment. Pharmacol. Ther..

[7] Akbarian A., Michiels J., Degroote J., Majdeddin M., Golian A., De Smet S. (2016). Association between heat stress and oxidative stress in poultry; mitochondrial dysfunction and dietary interventions with phytochemicals. J. Anim. Sci. Biotechnol..

[8] Estevez M. (2015). Oxidative damage to poultry: From farm to fork. Poult. Sci..

[9] Ma G., Chai X., Hou G., Zhao F., Meng Q. (2022). Phytochemistry, bioactivities and future prospects of mulberry leaves: A review. Food Chem..

[10] Kwon H.J., Chung J.Y., Kim J.Y., Kwon O. (2011). Comparison of 1-Deoxynojirimycin and Aqueous Mulberry Leaf Extract with Emphasis on Postprandial Hypoglycemic Effects: In Vivo and in Vitro Studies. J. Agric. Food Chem..

[11] Jiang L., Zhang L., Yang J., Shi H., Zhu H., Zhai M., Lu L., Wang X., Li X.Y., Yu S. (2022). 1-Deoxynojirimycin attenuates septic cardiomyopathy by regulating oxidative stress, apoptosis, and inflammation via the JAK2/STAT6 signaling pathway. Biomed. Pharmacother..

[12] Perera N., Brun J., Alonzi D.S., Tyrrell B.E., Miller J.L., Zitzmann N. (2022). Antiviral effects of deoxynojirimycin (DNJ)-based iminosugars in dengue virus-infected primary dendritic cells. Antivir. Res..

[13] Liu Q., Li X., Li C., Zheng Y., Wang F., Li H., Peng G. (2016). 1-Deoxynojirimycin Alleviates Liver Injury and Improves Hepatic Glucose Metabolism in *db*/*db* Mice. Molecules.

[14] Wang M., Feng Y., Li T., Zhao C., Barcenas A.R., Serrano B.R., Qu L., Shen M., Zhao W. (2023). The Effects of 1-Deoxynojirimycin from Mulberry on Oxidative Stress and Inflammation in Laying Hens and the Direct Effects on Intestine Epithelium Cells In Vitro. Animals.

[15] Chen Y., Wang J. (2024). 1-Deoxynojirimycin Attenuates High-Glucose-Induced Oxidative DNA Damage via Activating NRF2/OGG1 Signaling. Appl. Sci..

[16] Piao X., Li S., Sui X., Guo L., Liu X., Li H., Gao L., Cai S., Li Y., Wang T. (2018). 1-Deoxynojirimycin (DNJ) Ameliorates Indomethacin-Induced Gastric Ulcer in Mice by Affecting NF-kappaB Signaling Pathway. Front. Pharmacol..

[17] Ma Y., Lv W., Gu Y., Yu S. (2019). 1-Deoxynojirimycin in Mulberry (*Morus indica* L.) Leaves Ameliorates Stable Angina Pectoris in Patients with Coronary Heart Disease by Improving Antioxidant and Anti-inflammatory Capacities. Front. Pharmacol..

[18] National Research Council (1994). Nutrient Requirements of Poultry. Nutrient Requeriments of Domestic Animals.

[19] Chen X., Zhang L., Li J., Gao F., Zhou G. (2017). Hydrogen Peroxide-Induced Change in Meat Quality of the Breast Muscle of Broilers Is Mediated by ROS Generation, Apoptosis, and Autophagy in the NF-κB Signal Pathway. J. Agric. Food Chem..

[20] (2004). Feeding Standard of Chicken.

[21] Zhang S. (2023). From Challenge to Opportunity: Addressing Oxidative Stress in Animal Husbandry. Antioxidants.

[22] Yin L., Yang Q., Zhang Y., Wan D., Yin Y., Wang Q., Huang J., Li J., Yang H., Yin Y. (2021). Dietary Copper Improves Intestinal Morphology via Modulating Intestinal Stem Cell Activity in Pigs. Animals.

[23] Sun L., Xu G., Dong Y., Li M., Yang L., Lu W. (2020). Quercetin Protects against Lipopolysaccharide-Induced Intestinal Oxidative Stress in Broiler Chickens through Activation of Nrf2 Pathway. Molecules.

[24] Khan I., Zaneb H., Masood S., Yousaf M.S., Rehman H.F., Rehman H. (2017). Effect of *Moringa oleifera* leaf powder supplementation on growth performance and intestinal morphology in broiler chickens. J. Anim. Physiol. Anim. Nutr..

[25] Akib M.G., Rifat A., Bormon C., Dutta A., Ataher M.S., Azzam M., Farouk M.H., Das R., Azad M.A.K., Mahfuz S. (2024). Effects of *Moringa oleifera* Leaf Powder on the Growth Performance, Meat Quality, Blood Parameters, and Cecal Bacteria of Broilers. Vet. Sci..

[26] Anagnostopoulos E.C., Brouklogiannis I.P., Griela E., Paraskeuas V.V., Mountzouris K.C. (2023). Phytogenic Effects on Layer Production Performance and Cytoprotective Response in the Duodenum. Animals.

[27] Aguzey H.A., Gao Z., Wu H., Cheng G., Wu Z., Chen J. (2019). The effects of deoxynivalenol (don) on the gut microbiota, morphology and immune system of chicken—A review. Ann. Anim. Sci..

[28] Thakur K., Zhang Y.Y., Mocan A., Zhang F., Zhang J.G., Wei Z.J. (2019). 1-Deoxynojirimycin, its potential for management of non-communicable metabolic diseases. Trends Food Sci. Technol..

[29] Zheng J., Zhu L., Hu B., Zou X., Hu H., Zhang Z., Jiang N., Ma J., Yang H., Liu H. (2019). 1-Deoxynojirimycin improves high fat diet-induced nonalcoholic steatohepatitis by restoring gut dysbiosis. J. Nutr. Biochem..

[30] Liu Q., Li X., Li C., Zheng Y., Peng G. (2015). 1-Deoxynojirimycin Alleviates Insulin Resistance via Activation of Insulin Signaling PI3K/AKT Pathway in Skeletal Muscle of *db*/*db* Mice. Molecules.

[31] Takasu S., Parida I.S., Ito J., Kojima Y., Eitsuka T., Kimura T., Nakagawa K. (2020). Intestinal Absorption and Tissue Distribution of Aza-Sugars from Mulberry Leaves and Evaluation of Their Transport by Sugar Transporters. J. Agric. Food Chem..

[32] Vichasilp C., Nakagawa K., Sookwong P., Higuchi O., Luemunkong S., Miyazawa T. (2012). Development of high 1-deoxynojirimycin (DNJ) content mulberry tea and use of response surface methodology to optimize tea-making conditions for highest DNJ extraction. Lwt-Food Sci. Technol..

[33] Suzuki T. (2020). Regulation of the intestinal barrier by nutrients: The role of tight junctions. Anim. Sci. J..

[34] Jong C.J., Sandal P., Schaffer S.W. (2021). The Role of Taurine in Mitochondria Health: More Than Just an Antioxidant. Molecules.

[35] Zhuang Q., Guo F., Fu L., Dong Y., Xie S., Ding X., Hu S., Zhou X.D., Jiang Y., Zhou H. (2023). 1-Deoxynojirimycin promotes cardiac function and rescues mitochondrial cristae in mitochondrial hypertrophic cardiomyopathy. J. Clin. Investig..

[36] Kuo W.-T., Odenwald M.A., Turner J.R., Zuo L. (2022). Tight junction proteins occludin and ZO-1 as regulators of epithelial proliferation and survival. Ann. N. Y. Acad. Sci..

[37] Villanacci V., Del Sordo R., Lanzarotto F., Ricci C., Sidoni A., Manenti S., Mino S., Bugatti M., Bassotti G. (2024). Claudin-2: A marker for a better evaluation of histological mucosal healing in inflammatory bowel diseases. Dig. Liver Dis. Off. J. Ital. Soc. Gastroenterol. Ital. Assoc. Study Liver.

[38] Kang Y., Park H., Choe B.H., Kang B. (2022). The Role and Function of Mucins and Its Relationship to Inflammatory Bowel Disease. Front. Med..

[39] Liu Y., Yu X., Zhao J., Zhang H., Zhai Q., Chen W. (2020). The role of MUC2 mucin in intestinal homeostasis and the impact of dietary components on MUC2 expression. Int. J. Biol. Macromol..

[40] Daneshmand A., Kermanshahi H., Sekhavati M.H., Javadmanesh A., Ahmadian M. (2019). Antimicrobial peptide, cLF36, affects performance and intestinal morphology, microflora, junctional proteins, and immune cells in broilers challenged with *E. coli*. Sci. Rep..

[41] Jing M., Han G., Wan J., Zhang S., Yang J., Zong W., Niu Q., Liu R. (2020). Catalase and superoxide dismutase response and the underlying molecular mechanism for naphthalene. Sci. Total Environ..

[42] Sun Y., Wang X., Li L., Zhong C., Zhang Y., Yang X., Li M., Yang C. (2024). The role of gut microbiota in intestinal disease: From an oxidative stress perspective. Front. Microbiol..

[43] Hou Q., Qian Z., Wu P., Shen M., Li L., Zhao W. (2020). 1-Deoxynojirimycin from mulberry leaves changes gut digestion and microbiota composition in geese. Poult. Sci..

[44] Zhang D.D. (2010). The Nrf2-Keap1-ARE Signaling Pathway: The Regulation and Dual Function of Nrf2 in Cancer. Antioxid. Redox Signal..

[45] Thiruvengadam M., Venkidasamy B., Subramanian U., Samynathan R., Ali Shariati M., Rebezov M., Girish S., Thangavel S., Dhanapal A.R., Fedoseeva N. (2021). Bioactive Compounds in Oxidative Stress-Mediated Diseases: Targeting the NRF2/ARE Signaling Pathway and Epigenetic Regulation. Antioxidants.

[46] Suzuki T., Takahashi J., Yamamoto M. (2023). Molecular Basis of the KEAP1-NRF2 Signaling Pathway. Mol. Cells.

[47] Rex J., Lutz A., Faletti L.E., Albrecht U., Thomas M., Bode J.G., Borner C., Sawodny O., Merfort I. (2019). IL-1β and TNFα Differentially Influence NF-κB Activity and FasL-Induced Apoptosis in Primary Murine Hepatocytes During LPS-Induced Inflammation. Front. Physiol..

[48] Wang Y., Chen Y., Zhang X., Lu Y., Chen H. (2020). New insights in intestinal oxidative stress damage and the health intervention effects of nutrients: A review. J. Funct. Foods.

[49] Tang L., Xu Y., He J., Huang G., Jiang X., Li Y., Li H., Zhang R., Gui Z. (2024). 1-Deoxynojirimycin Derivative Containing Tegafur Induced HCT-116 Cell Apoptosis through Mitochondrial Dysfunction and Oxidative Stress Pathway. ACS Med. Chem. Lett..

